# Primitive neuroectodermal tumor of the pericardium: a case report and literature review

**DOI:** 10.1186/s12872-021-02113-3

**Published:** 2021-06-16

**Authors:** Jingjing Wang, Jing Li, Xiao Zhang, Xiaobo Zhang, Yueyong Xiao

**Affiliations:** 1 Department of Intensive Care Unit, Tianjin First Center Hospital, No.24 Fukang Street of Nankai District, Tianjin, 300152 China; 2 Department of Radiology, The First Medical Center of Chinese, PLA General Hospital, No.28 Fuxing Street of Haidian District, Beijing, 100853 China; 3 Department of MRI, Affiliated Hospital, Logistics University of Chinese People’s Armed Police Forces, Tianjin, 300162 China

**Keywords:** Primitive neuroectodermal tumor, Cardiac tumor, Prognosis, Case report

## Abstract

**Background:**

The primitive neuroectodermal tumors (PNETs) are a family of highly malignant tumors with a multidirectional differential potential. The tumors are characterized by aggressive small round tumor cells that originate from the spinal cord of the central and sympathetic nervous systems. Cases involving the pericardium are extremely rare. Herein, we present a case of peripheral primitive neuroectodermal tumor (pPNET) that originated in the pericardium.

**Case presentation:**

A 23-year-old woman presented with cough and progressive dyspnea for 1 month, followed by eyelid and facial edema for 10 days, without any apparent cause. Significantly elevated tumor markers were detected in her blood. A cardiac ultrasound revealed a 74 mm × 61 mm spherical mass that was attached to the left pericardium, as well as massive pericardial effusion. Positron emission tomography-CT (PET-CT) showed focal hypermetabolism in the left pericardium. Via histopathology and immunohistochemistry, the spherical mass was identified as PNETS. The patient was successfully treated with a combination of surgical resection via thoracotomy and postoperative chemotherapy, and she was disease-free for 7 years at follow-up. Unfortunately, at 7 years after the treatment, the patient’s pPNET recurred. Positron emission tomography-MRI (PET-MRI) and 64-slice coronary CTA revealed that the aorta and multiple coronary arteries were involved. Subsequently, the patient refused a heart transplant and voluntarily left the hospital.

**Conclusions:**

This paper reports on a rare and recurrent case of PNET in the parietal pericardium. With respect to the different biologic characteristics and prognoses of pPNETs (compared to other known pericardium tumors), it is essential to consider this entity as a differential diagnosis in pericardium tumors.

## Background

PNETs (primitive neuroectodermal tumors) are a family of highly malignant neoplasms that are characterized by small round cells of a neuroepithelial origin [1]. They usually involve the bone and soft tissues, and they have a higher incidence in childhood [2]. PNETs were first described in 1973 as a group of small round cell tumors that arise from mesenchymal progenitor cells, with the tumors belonging to a spectrum of neoplastic diseases known as the Ewing family of tumors (EFTs) [3]. Thus far, cases of different visceral locations of PNETs have been reported in the kidney, urinary bladder, ureter, prostate, penis, seminal vesicle, testis, small bowel, rectum, liver, gallbladder, pancreas, maxillary sinus, trachea, lung, heart, parotid gland, vulva, vagina, ovary, uterine cervix, uterus, breast and adrenal gland [4]. The pericardium is a rare primary location of PNETs. Here we report a case of a 23-year-old female patient with a recurrent pericardium pPNET for the first time.

## Case presentation

A 23-year-old female patient, healthy in the past, suffered from cough and post-exercising dyspnea for 1 month without any apparent cause. When her body position was changed, there was a sense of object movement in the precordial region of the heart. Ten days later, progressive dyspnea had originated, which was accompanied by eyelid and facial edema, and the patient was unable to lie on her back. A physical examination revealed bilateral cardiac enlargement with muffled heart sounds.

**Fig. 1:**
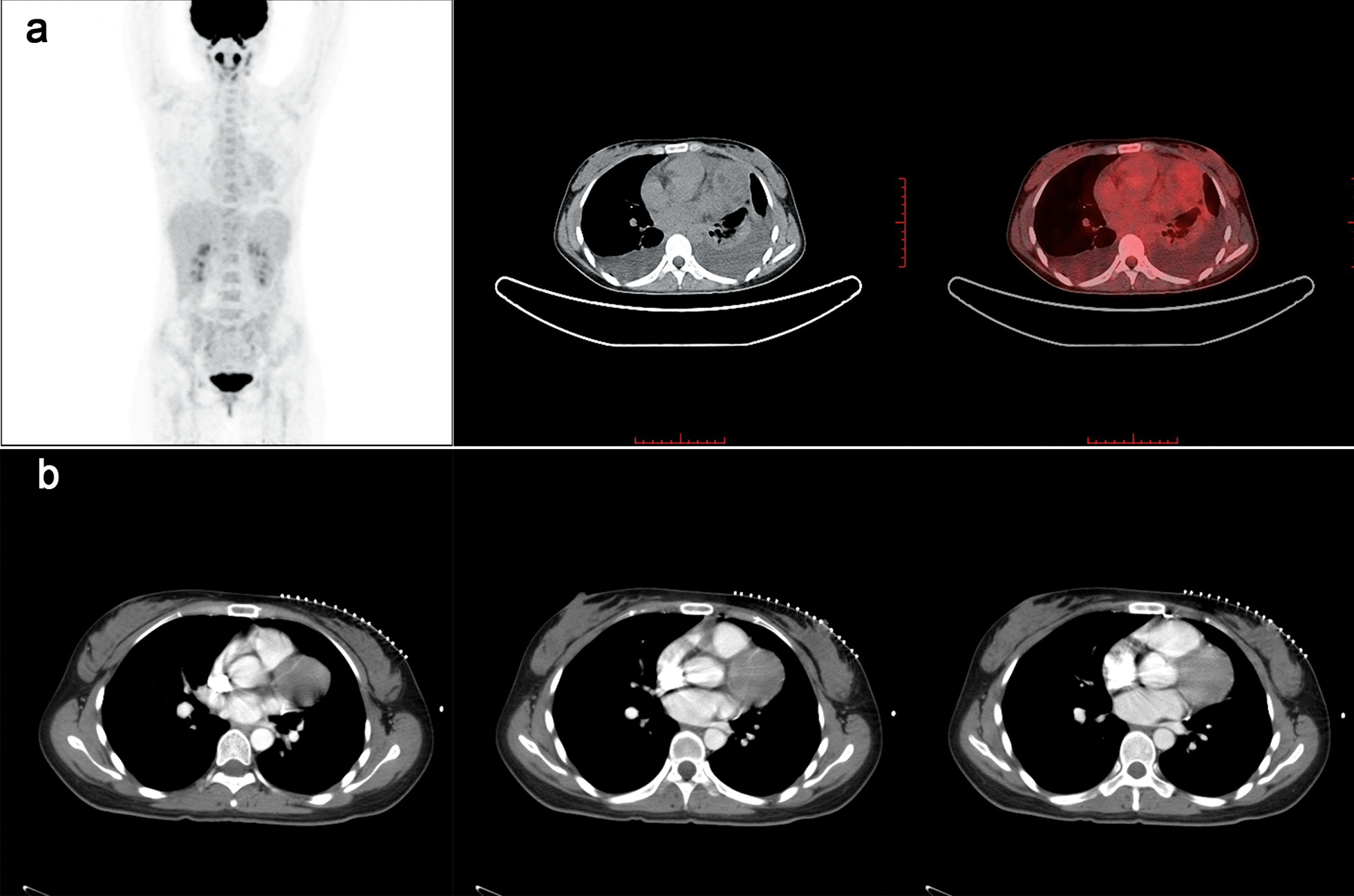
The imaging characteristics of the lesion before PNET excision. **a** Positron emission tomography-CT shows a soft tissue density with heterogeneous hypermetabolism. **b** Contrast-enhanced CT scan of the mediastinum, the mass in the left parietal pericardium showed no significant enhancements

**Fig. 2:**
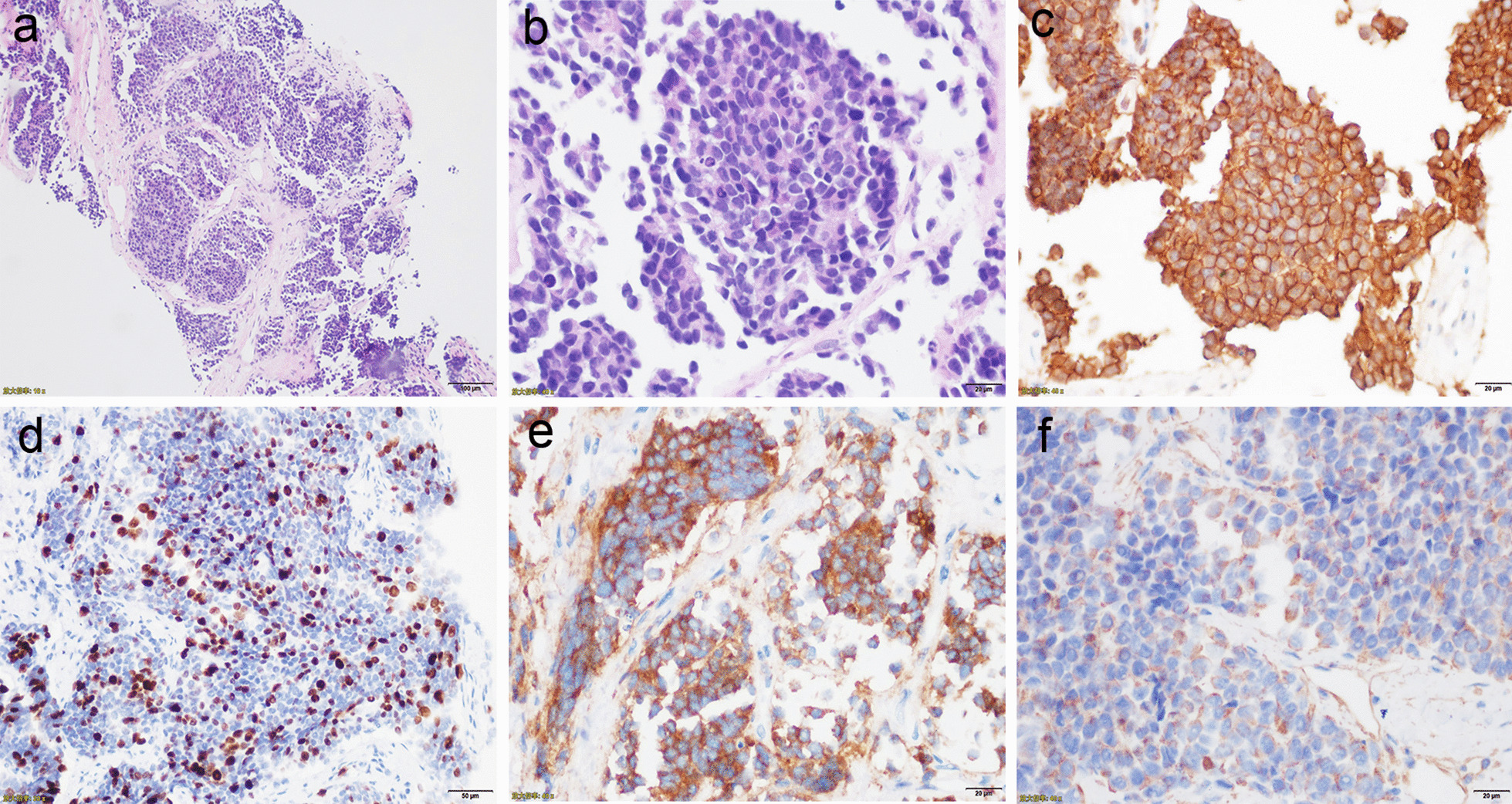
The histopathological characteristics of the tumor in the pericardium. **a**, **b** Hematoxylin and eosin staining. The higher power view shows uniform small round blue cells with scant cytoplasm. **c**–**f** Immunohistochemical staining (400 × magnification) of CD99, Ki67, NSE and Vimentin, respectively

A cardiac ultrasound revealed a 74 mm × 61 mm spherical mass that was attached to the left pericardium (the position of the mass did not change with the movement of the heart), as well as massive pericardial effusion. The PET-CT showed solid and mixed cystic lesions with low grade increased 18F-fluorodeoxyglucose (18F-FDG) uptake in the left parietal pericardium, and there were no signs of abnormal metabolism in other organs and tissues (Fig. 1a). On the contrast-enhanced CT scan of the mediastinum, the mass in the left parietal pericardium and in the left chest did not present significant abnormal enhancements (Fig. 1b). A significantly elevated tumor marker (CA-125, 298 U/ml) was detected in the blood. Subsequently, the patient received ultrasound-guided pericardiocentesis. The drainage was represented as a hemorrhagic effusion. The pericardial effusion analysis showed that fluid specific gravity was 1.043, ratio of pleural fluid to serum LDH > 0.6, ration of pleural fluid protein to serum protein > 0.5 (Table 1). According to Light’s criteria, pericardial effusion was exudative effusion. Additionally, the following multiple tumor markers in the pericardial effusion were observed to have doubled: CA-125 (864.5 U/ml), CA-199 (863.6 U/ml), CYFRA 21 (113.65 ng/ml), SCC (6.5 ng/ml) and serum ferritin (> 2000 ng/ml) (Table 2). The pericardial effusion analysis, and elevated level of multiple tumor markers in pericardial effusion were suggestive of malignant pericardial effusion. Afterwards, a pericardium puncture biopsy (under the guidance of ultrasound) was performed. The histological examination revealed a small round cell malignant tumor (Fig. 2a, b). Immunohistochemistry was positive for CD99 NSE, Vimentin, and the Ki-67 index was 30% (Fig. 2c–f). However, results for p63, CK and LCA were negative.

**Table 1:** Routine examination and biochemistry of the patient’s pericardial effusion

	Measured value	Normal range
Specific gravity	1.043	< 1.016
Protein	42 g/L	< 30 g/L
LDH	685U/L	< 200U/L
Protein of pericardial effusion/serum protein^a^	0.62	
LDH of pericardial effusion/serum LDH^b^	3.04	
Total cell number	1899.271 × 10^6^/L	< 100 × 10^6^/L
Ratio of polymorphocyte	0.24	
Ration of mononuclear cell	0.76	

^a^SERUM protein of the patient: 67.7 g/L

^b^Serum LDH of the patient: 225.3 U/L

**Table 2:** The concentration of tumor markers in the patient's pericardial effusion

	Concentration	Normal range
CA-125	864.5 U/mL	0.1–35 U/mL
CA 19-9	863.6 U/mL	0.1–37 U/mL
CYFRA21-1	13.65 ng/mL	0.1–4.0 ng/mL
SCC	6.5 ng/mL	< 1.8 ng/mL
Serum ferritin	> 2000 ng/mL	13–150 ng/mL

**Fig. 3:**
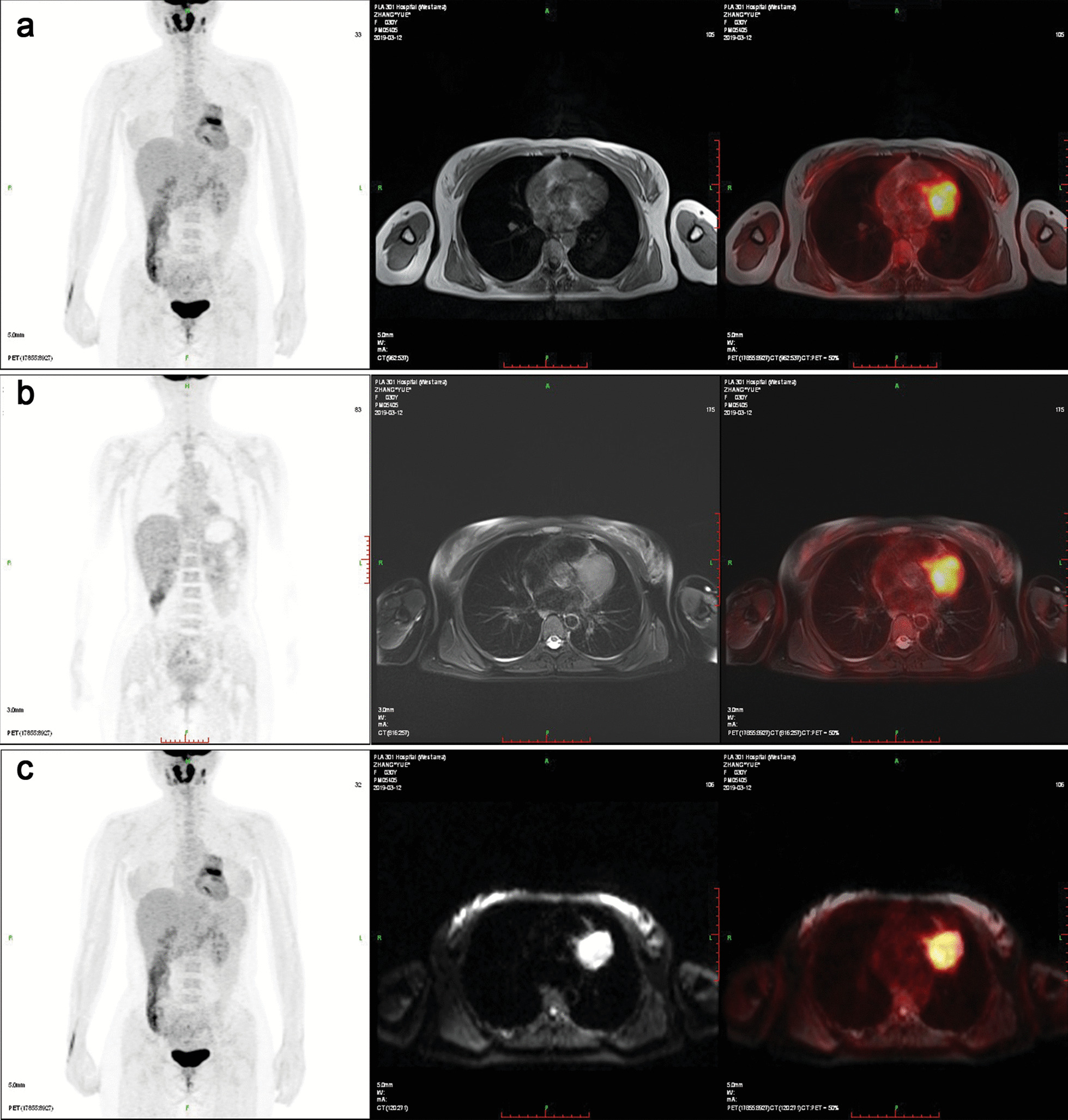
The imaging characteristics of PNET on PET/MRI at recurence. Positron emission tomography-MRI shows an irregular soft-tissue mass in the pericardial cavity with apparent higher metabolism. **a** The lesions showed iso-intense signals on T1WI. **b** The lesions showed hyperintense signals on T2WI. **c** The lesions showed significantly inhomogeneous hyperintense signals on the DWI

**Fig. 4:**
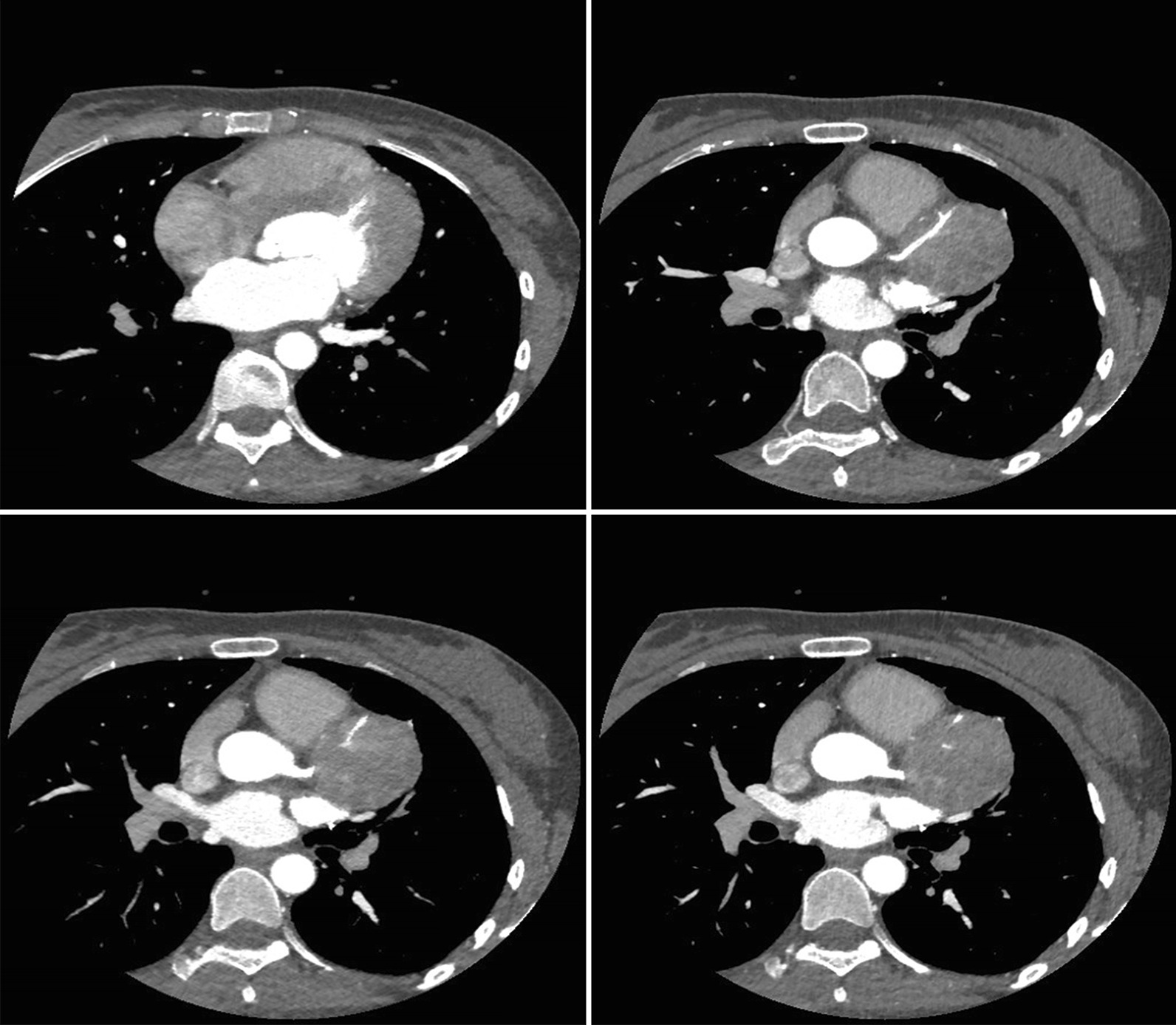
The imaging characteristics of PNET on coronary CTA at recurrence. 64-slice coronary CTA showed a solid and cystic lesion in the pericardial cavity, which circled the coronary artery

Ten days later, the patient received a tumor excision procedure via open-chest surgery, followed by four courses of chemotherapy every 3 weeks. The patient received the standard chemotherapy consisted of 2 mg of vincristine per square meter of body surface area, a bolus injection of doxorubicin at a dose of 75 mg per square meter of body surface area, and 1200 mg of cyclophosphamide per square meter [5]. Based on the surgical findings and the histopathology, this case was diagnosed as a PNET of the pericardium. Seven years after the surgery, the patient’s PNET in the pericardium recurred. A PET-MRI showed a 4.6 cm × 4.3 cm mass in the original operating area, which involved the aortic root. The lesions showed iso-intense signals on the T1WI (Fig. 3a) and hyperintense signals on the T2WI (Fig. 3b). The lesions showed significantly inhomogeneous hyperintense signals on the DWI (Fig. 3c). A 64-slice coronary CTA revealed that the tumor pulled and circled the proximal-middle segments of the left anterior descending branch of the coronary artery (Fig. 4). In this situation, the patient did not have surgical indications for a local excision. Her only chance of survival was a heart transplant. Unfortunately, the patient refused this treatment option and voluntarily left the hospital.

## Discussion and conclusion

Primary cardiac tumors are unusual. PNETs of the heart are extremely rare malignancies. To our knowledge, there have been 9 reported cases of primary cardiac PNETs. Arterial and ventricular locations are the common sites [6]. So far, only three cases of PNETs in the pericardium has been reported [7]. Our case was the fourth case in this literature. Most of the reported PNET cases were male [8]. Our patient was a female. Consistently, primary PNETs of the pericardium exhibits female predominance (3 females and 1 male having reported PNETs in the pericardium) [7].

PNETs are classified into central primitive neuroepidermal tumors (cPNETs) and peripheral primitive neuroepidermal tumors (pPNETs) [9]. pPNETs usually occur in the sympathetic nervous system and bone [10]. Heart is innervated by the sympathetic and parasympathetic nervous systems. Therefore, heart can be a possible location of a pPNETs. The symptoms of cardiac primary or metastatic tumors are nonspecific and depend on the location of the tumor. Our patient had a history of exertional dyspnea and a blockage of the superior vena cava blood return. Transthoracic echocardiography is the first choice for the initial diagnosis. CT and MRI scans play a main role in diagnosis and evaluation of the local invasion and distant metastasis [6]. Consistently with previous reports [1], the tumor in this present study on CT/MR image was rounded masses with well-defined margins. Solid and mixed cystic changes of the tumor were related to inadequate blood supply to the corresponding region in the tumor. PET-CT used in the first occurrence of this patient was due to the advantage of PET-CT/PET-MRI could detect the distant metastasis [11]. PET-CT is much better in detecting lung metastasis, and PET-MRI is much better in detecting liver as well as bone metastasis [11]. These technologies help clinicians to make the clinical decision.

PNETs are rare, small round cell malignancies, which originate from the neural crest cells. In the current case, the tumor in the pericardium biopsy initially required a number of differential diagnostic considerations. Other types of pericardium tumors include mesothelioma. Immunohistochemistry is the only method to make a definitive diagnosis. Mesothelioma is an epithelioid malignant tumor, which is confirmed by positive indications of mesothelial markers (calretinin, WT-1, cytokeratin 5/6 and D2-40) [12]. The CD99 marker is positive in rhabdomyosarcoma and synovial sarcoma. Conversely, (11; 22) (q24; q12) translocation is specific to PNETs [13]. CD99, CD56, cytokeratin, GFAP, NSE and synaptophysin markers are indicative of neuroectodermal cells [14]. CD99, accompanied by NSE, is the currently most accepted immunohistochemical marker for pPNETs [15]. The presence of Homer-Wright Rosette structures and CD99, combined with a positive NSE marker, in our case indicates that the patient had pPNETs.

pPNETs are extremely aggressive and lethal. The survival rate is 25% for tumors that are larger than 5 cm at 24 months [16]. Treatments of PNETs require a combination of surgery, chemotherapy and radiotherapy. Compared to the surgical excision alone, a combination therapy improves the 2-year survival rate from 23–44 to 59–67%, and the distant metastasis rate can decrease from 46–65 to 12–13% [17]. Whether the primary site is completely resected or not can affect the prognosis of patients with PNETs [17]. Strangely, the progression free survival of the patient in our case was approximately 7 years following the surgery combined with chemotherapy.

Primary cardiac and pericardial neoplasms, as well as pPNETs, are both rare tumors. The primary pericardial PNETs is especially rare. The diagnosis of this condition depends not only on the histopathology but also on obtaining reliable tissue via image-guided location procedures. Therefore, when patients present with cardiac or pericardial tumors, PNETs should be considered as a differential diagnosis.

## Data Availability

Not applicable.

## Abbreviations

PNETs: Primitive neuroectodermal tumors
pPNETs: Peripheral primitive neuroectodermal tumors
PET-CT: Positron emission tomography-CT
PET-MRI: Positron emission tomography-MRI
EFTs: Ewing family of tumors
PNET: Primitive neuroectodermal tumor
cPNETs: Central primitive neuroepidermal tumors

## References

[1] Zhang Y, Cai P, Chen M, Yi X, Li L, Xiao D (2016). Imaging findings of adrenal primitive neroectodermal tumors: a seies of seven cases. Clin Transl Oncol.

[2] Rafael EJ, Andrew LF, Rosanna LL, Jae YR, Patricia AO, Sharon WW, Mahul BA (2002). Primary Ewing's sarcoma/primitive neuroectodermal tumor of the kidney: a clinicopathologic and immunohistochemical analysis of 11 cases. Am J Surg Pathol.

[3] Hart MN, Earle KM (1973). Primitive neuroectodermal tumors of the brain in children. Cancer.

[4] Koufopoulos N, Kokkali S, Manatakis D, Balalis D, Nasi D, Ardavanis A (2019). Primary peripheral neuroectodermal tumor (PNET) of the adrenal gland: a rare entity. J BUON.

[5] Grier HE, Krailo MD, Tarbell NJ, Link MP, Fryer CJH, Pritchard DJ (2003). Addition of ifosfamide and etoposide to standard chemotherapy for Ewing's sarcoma and primitive neuroectodermal tumor of bone. N Engl J Med.

[6] Rajappa S, Gundeti S, Varadpande L, Bethune N, Rao S, Digumarti R (2007). Cardiac primitive neuroectodermal tumor presenting as acute coronary syndrome. J Clin Oncol.

[7] Mohandas KM, Chinoy RF, Merchant NH, Lotliker RG, Desai PB (1992). Malignant small cell tumour (Askin-Rosai) of the pericardium. Postgrad Med J.

[8] Ng TL, O’Sullivan MJ, Pallen CJ, Hayes M, Clarkson PW, Winstanley M (2007). Ewing sarcoma with novel translocation t(2;16) producing an in-frame fusion of FUS and FEV. J Mol Diagn.

[9] Burkhardt JK, Kockro RA, Dohmen-Scheufler H, Woernle CM, Bellut D, Kollias S (2011). Small supratentorial, extraaxial primitive neuroectodermal tumor causing large intracerebral hematoma. Neurol Med Chir (Tokyo).

[10] Park JY, Lee S, Kang HJ, Kim HS, Park SY (2007). Primary Ewing's sarcoma-primitive neuroectodermal tumor of the uterus: a case report and literature review. Gynecol Oncol.

[11] Ming Y, Wu N, Qian T, Li X, Wan DQ, Li C (2020). Progress and future trends in PET/CT and PET/MRI molecular imaging approaches for breast cancer. Front Oncol.

[12] Arif Q, Husain AN (2015). Malignant mesothelioma diagnosis. Arch Pathol Lab Med.

[13] Fan C, Kong D, Tan C, Yang J (2017). Isolated cardiac peripheral primitive neuroectodermal tumor: a case report. Cancer Biol Ther.

[14] Chen Z, Chen R, Ng C, Shi H (2018). Primary pericardial primitive neuroectodermal tumor: a case report and review of literature. Int J Clin Exp Pathol.

[15] Tsokos M, Alaggio RD, Dehner LP, Dickman PS (2012). Ewing sarcoma/peripheral primitive neuroectodermal tumor and related tumors. Pediatr Dev Pathol.

[16] Kushner BH, Hajdu SI, Gulati SC, Erlandson RA, Exelby PR, Lieberman PH (1991). Extracranial primitive neuroectodermal tumors. The Memorial Sloan-Kettering Cancer Center experience. Cancer.

[17] Saeedinia S, Nouri M, Alimohammadi M, Moradi H, Amirjamshidi A (2012). Primary spinal extradural Ewing's sarcoma (primitive neuroectodermal tumor): report of a case and meta-analysis of the reported cases in the literature. Surg Neurol Int.

